# Formulation, Characterization and Properties of Hemp Seed Oil and Its Emulsions

**DOI:** 10.3390/molecules22050700

**Published:** 2017-04-27

**Authors:** Veronika Mikulcová, Věra Kašpárková, Petr Humpolíček, Leona Buňková

**Affiliations:** 1 Department of Fat, Surfactant and Cosmetics Technology, Faculty of Technology, Tomas Bata University in Zlin, nam. T. G. Masaryka 5555, 760 01 Zlin, Czech Republic; mikulcova@ft.utb.cz; 2 Centre of Polymer Systems, Tomas Bata University in Zlin, nam. T. G. Masaryka 5555, 760 01 Zlin, Czech Republic; humpolicek@ft.utb.cz; 3 Polymer Centre, Faculty of Technology, Tomas Bata University in Zlin, 760 01 Zlin, Czech Republic; 4 Department of Environmental Protection and Engineering, Faculty of Technology, Tomas Bata University in Zlin, nam. T. G. Masaryka 5555, 760 01 Zlin, Czech Republic; bunkova@ft.utb.cz

**Keywords:** hemp seed oil, emulsion, particle size, fatty acid composition, antibacterial activity

## Abstract

The formulation, characterization, and anticipated antibacterial properties of hemp seed oil and its emulsions were investigated. The oil obtained from the seeds of *Cannabis sativa* L. in refined and unrefined form was characterized using iodine, saponification, acid values, and gas chromatography, and was employed for the preparation of stable oil-in-water emulsions. The emulsions were prepared using pairs of non-ionic surfactants (Tween, Span). The effects of the emulsification method (spontaneous emulsification vs. high-intensity stirring), hydrophilic lipophilic balance (HLB), type and concentration of surfactant, and oil type on the size and distribution of the emulsion particles were investigated. It was found that the ability to form stable emulsions with small, initial particle sizes is primarily dependent on the given method of preparation and the HLB value. The most efficient method of emulsification that afforded the best emulsions with the smallest particles (151 ± 1 nm) comprised the high-energy method, and emulsions stable over the long-term were observed at HBL 9 with 10 wt % concentration of surfactants. Under high-intensity emulsification, refined and unrefined oils performed similarly. The oils as well as their emulsions were tested against the growth of selected bacteria using the disk diffusion and broth microdilution methods. The antibacterial effect of hemp seed oil was documented against *Micrococcus luteus* and *Staphylococcus aureus* subsp. *aureus*. The formulated emulsions did not exhibit the antibacterial activity that had been anticipated.

## 1. Introduction

Hemp seed oil, obtained from the seeds of *Cannabis sativa* L., is known for its nutritive, health-enhancing properties and bioactivity. Compared to other vegetable oils, it is an especially rich source of both n-3 and n-6 essential fatty acids, namely linoleic acid (18:2 n-6, at 55 wt %) and alpha-linolenic acid (18:3 n-3, at 20 wt %). The content of gamma-linolenic acid (18:3 n-6) equals approximately 1–4 wt %, while that of stearidonic acids (18:4 n-3) ranges from 0.5–2 wt % [1]. Of no less importance is that hemp seed oil contains a moderate to high amount of tocopherols and tocotrienols (100 to 150 mg per 100 g of oil), phytosterols, phospholipids, carotenes, and minerals [2]. Interestingly, the aforementioned beneficial properties of hemp seed oil offer numerous potential applications, e.g., as components in functional foodstuffs and in treating various health problems. Here, the lowering of high cholesterol and high blood pressure can be named as examples [3,4]. The health benefits of hemp seed oil are attributed mainly to its desirable n-3 and n-6 fatty acid ratio, 3:1, which is suggested as being optimal for human nutrition [5,6]. It has been shown that unbalanced intake of n-3 and n-6 fatty acids is associated with many diseases, e.g., diabetes, cardiovascular diseases, and cancer. The presence of gamma-linolenic acid in the oil, which is deficient in the average Western diet, is also noteworthy [6]. This unique composition of hemp seed oil differs from the other common seed oils and offers opportunities for the development of special nutritional formulations [7,8,9]. However, besides the well-documented health and nutrition effects, the antimicrobial and specifically the antibacterial effects of hemp seed oil still remain questionable [10,11].

Although many studies have focused on preparing oil-in-water (O/W) emulsions consisting of either mineral or synthetic oils, less attention has been paid to their formation in the presence of vegetable oils as a dispersed phase. Moreover, most studies and applications of vegetable oil-based emulsions involve one of the following commonly used oils; palm, soybean, rapeseed, sunflower, coconut, palm-kernel, cottonseed, groundnut, and olive [4,12,13]. However, with the development of the nutraceutical and functional foods market, more attention is now devoted to non-traditional vegetable oils and their encapsulation in emulsions. Examples include argan oil [14], pomegranate seed oil [15], and grapeseed and sesame oils [16]. In this context, the application of hemp seed oil in emulsions, which might further enhance its beneficial properties, has not been fully exploited. Due to reduced sizes of droplets and high surface to volume ratio, loading of the oil in nanoemulsions may improve the bioavailability of present unsaturated fatty acids [7], help to protect the oil against oxidation and interaction with other ingredients, and contribute as a dietary source of natural antioxidants for disease prevention and health promotion [9,17]. Only a few studies have actually dealt with hemp seed oil emulsions (water-in-oil) while, as far as the authors are aware, even less research has concentrated on oil-in-water emulsions [18,19].

The past few years have also witnessed a widening in the utilization of oils from plant sources into non-food areas, such as the pharmaceutical and cosmetics industries, due to their qualities of being non-toxic, biodegradable, and environmentally friendly. This is reflected in current research on the exploration of vegetable-oil-based emulsions [20,21]. The aim of this paper was to prepare stable oil-in-water emulsions based on hemp seed oil, and determine the influence of emulsion composition and preparation methods on their characteristics, including stability. In this regard, the quality of two brands of oil extracted from hemp seeds was investigated to describe differences between the properties of refined and unrefined oil when utilized in emulsions. Research was also carried out on the possible bioactivity of the hemp seed oils and their emulsions as pertaining to their antibacterial properties against common pathogens.

## 2. Results and Discussion

Prior to preparing the emulsions, basic characterization of the hemp seed oils was carried out in order to elucidate differences between their refined and unrefined types. The iodine values of 155.8 ± 1.9 and 167.4 ± 0.6 g iodine/100 g of oil were determined for refined and unrefined oil, respectively. Correspondingly, the saponification values of 197.6 ± 4.5 and 202.2 ± 3.9 mg KOH/g of oil, and acid values of 1.7 ± 0.6 and 0.7 ± 0.0 mg KOH/g of oil were measured. Comparing the obtained results with the data published by Anwar et al. [22] revealed reasonably good compliance. The published iodine values were quite similar to those herein, ranging from 154 to 165 g iodine/100 g oil and the saponification values were slightly lower (184–190 mg KOH/g of oil). The fatty acid composition of the oils is provided in Table 1. The main differences between the contents of particular fatty acids pertained to oleic and alpha-linolenic acids. Regarding the content of alpha-linolenic acid in unrefined and refined oils, values of 20.3 ± 0.03 wt % and 16.7 ± 0.04 wt %, respectively, were measured. The content of oleic acid was 12.1 ± 0.03 wt % and 9.0 ± 0.1 wt % for the unrefined and refined samples, respectively. However, the variations observed did not obviously deviate from the fatty acid contents reported for a range of hemp seed oils by different authors [22,23]. It is therefore apparent that oil composition prevailingly depends on its origin and/or the extraction procedure used to obtain it. Unrefined hemp seed oil also possessed trace concentrations of eicosadienoic and myristic acids.

### 2.1. Formulation of Emulsions

**Figure 1:**
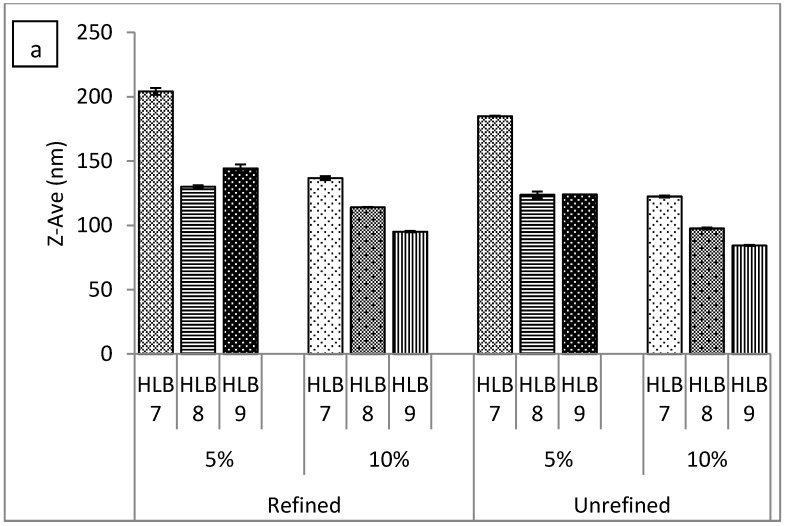

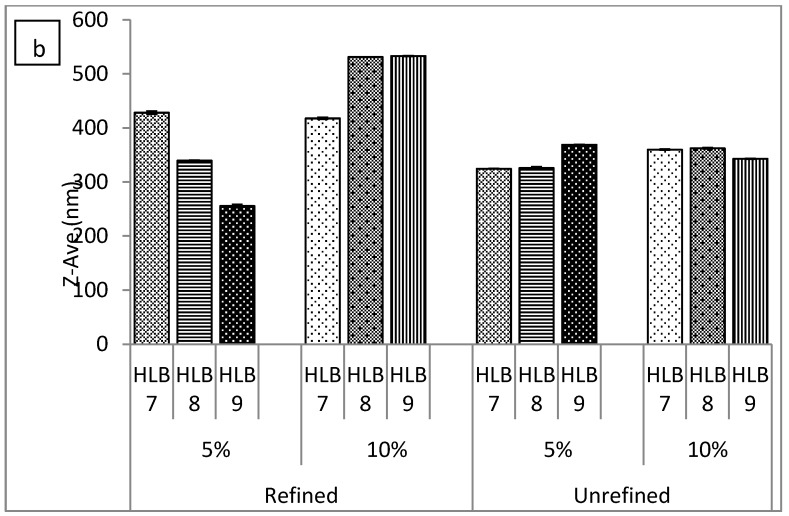
Effect of oil type (refined vs. unrefined), hydrophilic lipophilic balance (HLB), and surfactant concentration on the particle size of emulsions prepared by (**a**) high-energy method and (**b**) low-energy method. Tween 85/Span 85 ratios in the emulsions were 1.30 (HLB 7), 2.07 (HLB 8), and 3.60 (HLB 9).

The O/W emulsions were prepared by two different emulsification procedures; these also involved varying the types and concentrations of emulsifiers, with the hydrophilic-lipophilic balance (HLB) ranging from 6 to 10, as well as utilizing two types of hemp seed oil. Key characteristics of an emulsion are the size and distribution of the emulsion particles, as their changes indicate the stability of the formulation. As expected, it was discerned that particle size and distribution were notably influenced by the method of preparation. This is illustrated in Figure 1, which shows the particle size of emulsions and methods for preparing the same, relating to the samples based on Span 85/Tween 85.

**Figure 2:**
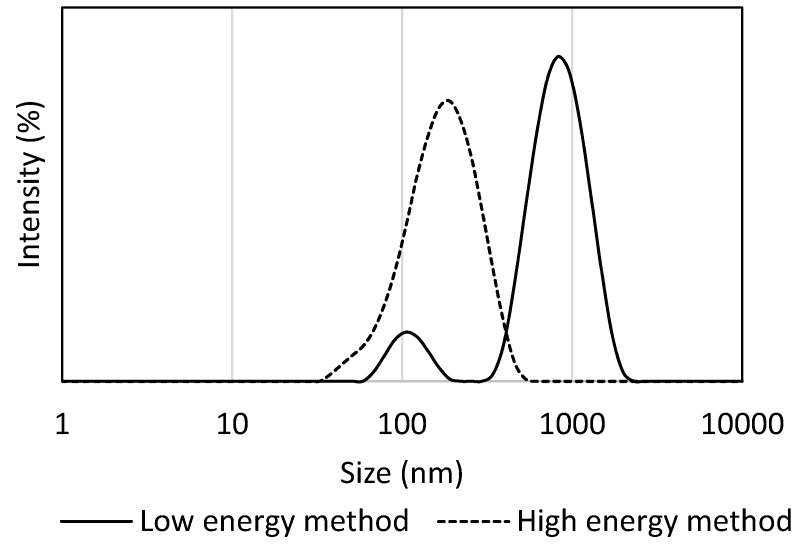
Particle size distribution recorded after emulsification with low-energy vs. high-energy conditions at emulsions with 5 wt % Tween 80/Span 80 and HLB 9. The Tween 80/Span 80 ratio was 0.78.

As can be seen, the diameters of the particles present in all emulsions prepared by the high-energy method were significantly smaller (*p* ≤ 0.001), regardless of HLB values, than the emulsions produced by the low-energy EIP (emulsion inversion point) method. This observation is in agreement with a study published by Ostertag et al. [24]. In this study, only relatively large droplets (>600 nm) were formulated by the EIP method using long-chain triglyceride oils (such as olive, grape seed, sesame, peanut, and canola oils). Similar findings were reported by Gullapalli and Sheth [25], who found that non-ionic emulsifiers reduced the particle size of hydrocarbon-in-water emulsions more effectively than triglyceride-in-water emulsions. Recently, exotic vegetable oil-in-water nanoemulsions with the addition of ethoxylated and acetylated lanolin have been obtained by using a low energy EIP method. It has been shown that the lanolin derivative addition caused alterations of droplet size and conductivity of the systems, however the droplet size remained still within the nanometer range (20–200 nm) [26]. Lane et al. [27] reported on flaxseed and algae nanoemulsions formulated with combinations of Tween 40 and lecithin. Stable emulsions were prepared up to 50 wt % of oil content with a droplet size of 192 nm for flaxseed-oil and 182 nm for emulsions loaded with algae oil. In the study of Krasodomska and Jungnickel [28], various seed oils (apple, strawberry, and raspberry) have been used as components of the oil phase in O/W emulsions. The best emulsion contained 4 and 5 wt % of seed oil together with other components of the oil phase and the O/W ratio was 20/80. The emulsification procedure also affected the particle size distribution of the emulsions, and homogenization via the high-energy method brought about emulsions with narrower distribution, in comparison with the EIP method. This is shown in Figure 2 for systems consisting of the Tween 80/Span 80 mixture (5 wt %) at HLB 9, where only the main particle population was observed when the high-energy method was utilized, while EIP afforded bimodal size distribution with the occurrence of two main droplet populations.

On average, emulsions prepared using the high-energy method contained considerably smaller droplets than systems prepared with the EIP technique. Immediately after preparation, the former of the two systems showed particles from 151 ± 1 nm to 209 ± 5 nm, whilst the particle sizes of the latter were significantly higher (*p* ≤ 0.001) and ranged from 502 ± 22 nm to 1050 ± 29 nm. Correspondingly, the polydispersity index (PDI) of the high-energy samples was approximately 0.18 ± 0.01 to 0.26 ± 0.01, whereas the PDI of the emulsions prepared by EIP ranged from 0.26 ± 0.09 to 0.75 ± 0.44. It is known that the PDI is an estimate of the width of the droplet distribution in samples. The PDI values of approximately 0.1 correspond to the polydispersity of monodisperse standards and values greater than 0.7 indicate that the sample has a very broad size distribution. The PDI values measured on the studied emulsions therefore clearly show that the high-energy method provides significantly narrower droplet distributions than the EIP technique.

### 2.2. Influence of HLB and Oil Type

**Figure 3:**
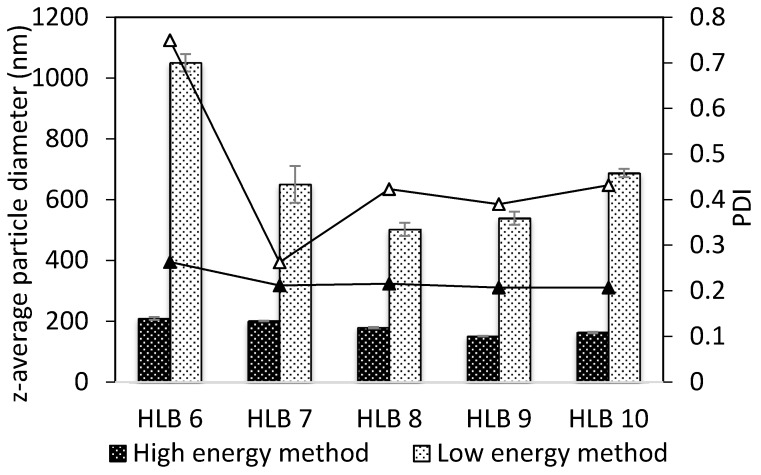
The influence of the HLB on particle sizes (columns) and polydispersity index (lines) of the nanoemulsions (5 wt % Tween 80/Span 80) prepared by the low-energy and high-energy methods using refined oil.

Finding the optimum HLB value required for the successful encapsulation of the hemp seed oil was based on the premise that at optimum HLB, the mean particle sizes of the emulsion droplets are at their minimum. This factor also influences, to a large extent, the stability of the emulsions produced. Another possible procedure to determine optimum HLB encompasses visual observation of the emulsions, and the system with minimal creaming and phase separation is deemed to possess the optimum HLB [29]. The values chosen for preparing the emulsions ranged from 6–10 for the pairs of Tween 80/Span 80 and 7–9 for Tween 85/Span 80. Figure 3 depicts the impact of the HLB value on the particle sizes of the emulsions, revealing the evolution of droplet size concurrent with changes in HLB for Tween 80/Span 80 under differing preparation conditions. This figure shows that the optimal HLB for hemp seed oil lies between 8 and 9, and is identified with affording the minimum average particle size, irrespective of the method used for producing the emulsions. In the case of the EIP method, a U-shaped curve is clearly visible, showing the strong correlation that exists between particle size and HLB. Unfortunately, despite finding the optimum HLB value for this system, it was not possible to formulate stable emulsions with EIP and emulsions prepared by this low-energy method became unstable or broke down within several minutes of preparation. On the contrary, for analogous emulsions prepared by the high-energy method, merely negligible changes in droplet size alongside changes in HLB were observed. Furthermore, the emulsions were reasonably stable, not exhibiting any sign of destabilization. In the literature, the aforementioned U-shape dependence of particle size on HLB is much less reported for the high-energy methods than the low-energy methods (such as EIP) [30]. Figure 3 also highlights the significant impact of HLB and the method of emulsion preparation on the PDI, which was systematically higher in emulsions prepared by EIP. Hence, the low-energy method utilized is far more sensitive to the proper choice of emulsion composition than the high-energy method. Of all the samples, the smallest particles (84 ± 1 to 122 ± 2 nm) were achieved at any given HLB with the Tween 85/Span 85-based emulsions (10 wt %) prepared by the high-energy method.

The above results demonstrated that emulsions with fine droplets could primarily be produced by the high-energy approach, but the question remained as to whether the type of oil (refined vs. unrefined) actually influenced the particle sizes of emulsions formulated through both approaches. Figure 1a clearly shows the similar behaviour exhibited by the Tween 85/Span 85 emulsions over the range of the HLB tested, irrespective of the type of oil used when applying the high-energy method. This contrasted with emulsions prepared by the EIP method (Figure 1b), which tended to vary and behave differently, even if they shared the exact same formulation. Here, a statistically significant difference (*p* ≤ 0.01) was observed for particle sizes of the emulsions formulated with refined and unrefined oils, respectively. It was also seen that higher concentrations of surfactant provided more uniform systems across the HLB range examined and enabled the preparation of emulsions with significantly smaller particles (*p* ≤ 0.01).

### 2.3. Emulsion Stability

The long-term stability of the hemp seed oil-in-water emulsions was assessed over a period of a maximum of 230 days by recording changes in their visual appearance during storage at 25 and4 °C. The best stability discerned for emulsions formed by the EIP method, regarding the visual assessment, was demonstrated by emulsions containing 10 wt % Tween 85/Span 85 at HLB 7. These emulsions were stable for 8 days and such early break-down was attributed to their relatively large sizes of particles (590 to 780 nm). However, emulsions prepared on the Ultraturrax device (IKA, Staufen, Germany), stabilized by Tween 80/Span 80, were stable for 24 days of storage, with no phase separation or creaming observed. Later, when creaming of the emulsions was recorded, no significant changes were observed in particle size, as measured by dynamic light scattering (DLS). The greatest stability was shown by Tween 80/Span 80-based systems at HBL 9 and 10 wt % surfactant (stable for 230 days), and the Tween 85/Span 85 system (200 days), stored at both temperatures. This long-term stability might be associated with the small, initial droplet size observed after preparation. According to study conducted by Rao and McClements [31], lemon oil nanoemulsions with initially smaller droplets were more stable against coalescence, aggregation, as well as flocculation. Similar findings were also reported in several other papers. For example, in recent works studying the effect of various processing factors on the formulation of vegetable oil-based nanoemulsions [32,33], the smaller droplets resulted in their better long-term stability, which also correlated well with values of droplet sizes predicted by using mathematical modelling.

**Figure 4:**
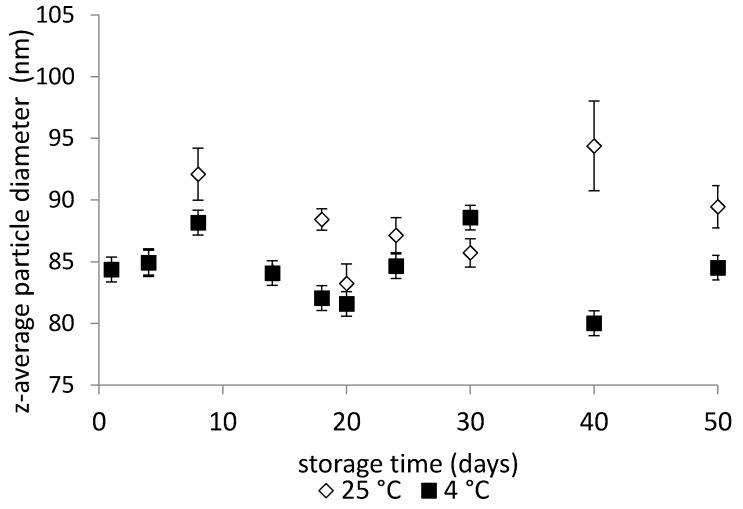
Changes in the z-average diameter of emulsion droplets as a function of storage time and temperature (4 and 25 °C) determined for emulsions with Tween 85/Span 85 (10 wt %) at HLB 9.

Variations in mean particle sizes during the storage of emulsions formulated with Tween 85/Span 85 (10 wt %) at HLB 9 are illustrated in Figure 4, which documents only minor changes, regardless of the storage temperature. This finding is consistent with the study of Rebolleda et al. [34], in which wheat bran oil nanoemulsions stabilized by mixtures of Span 80/Tween 80 showed only negligible changes in the droplet size of the emulsions during storage.

### 2.4. Antibacterial Activity

Both hemp seed oils and their emulsions were screened with respect to their antibacterial effects. Table 2 displays the results from the disc diffusion method as recorded for both oils, revealing their weak antibacterial activity against selected species. Using this method, the antibacterial activity was determined by the measurement of the diameter of the inhibitory zone (mm) formed around the discs soaked with the oil and the size of the disc was subtracted from the size of the inhibitory zone. The unrefined oil possessed inhibition to all strains, with inhibition zones ranging from 0.3 to 3.3 mm, and the effect was mainly dependent on the type of bacteria. These results were further supported by a broth microdilution method (data not presented). A previous report on the antimicrobial activity of hemp seed oil was published by Leizer et al. [11], who observed some bioactivity during primary screening. In past years, the antibacterial activity of essential oils extracted from *C. sativa* was, however, more extensively studied [35,36].

In order to understand the difference in antibacterial activity of both types of oil, the results from antibacterial testing were compared to the fatty acid analyses. As stated earlier, the antibacterial action of fatty acids is usually recognized for long-chain unsaturated fatty acids, including oleic acid, linoleic acid, and linolenic acid [37]. The higher content of alpha-linolenic acid in the unrefined oil could explain the increase in antibacterial activity, compared with findings for the refined oil. Another possible explanation might pertain to the fact that during the refining process, minor components such as tocopherols and tetrahydrocannabinol are removed, which may contribute to the antibacterial activity of unrefined hemp seed oils [38].

As reported earlier [39], gram-positive bacteria were seen to be more sensitive to the unrefined oil than gram-negative strains, as a result of differences in the composition of the bacterial cell wall. In accordance with this fact, *E. coli* was the most resistant species, and *M. luteus* and *S. aureus* proved to be most sensitive to the oils tested. However, statistical analysis did not prove any significant difference between the group of gram positive and gram negative strains regarding the effect of refined and unrefined oils, respectively. Though the observed antibacterial activity of hemp seed oil was weak, it can be regarded as an added value to the main positive characteristics of hemp seed oil, namely the content of n-3 and n-6 fatty acids. Additionally, an investigation was conducted on the potential antibacterial effects of the formulated emulsions. Previously, a synergistic effect of antibacterial substances encapsulated in emulsions or nanoemulsions has been observed, as reported by Ghosh et al. [40]. The enhanced activity of emulsions against microorganisms is primarily explained by the presence of non-ionic surfactants in the formulations. Several studies [30,41] reported on increased antibacterial activity when non-ionic Tween 80 made up part of the formulation. Therefore, a similar effect was expected for the hemp seed oil emulsions studied herein. Nevertheless, the disk diffusion and broth dilution methods did not reveal the rise that had been anticipated in antibacterial activity by emulsions containing oil, relative to pure forms of oils alone.

## 3. Experimental Methods

### 3.1. Materials

Two types of cold-pressed hemp seed oil were employed: an unrefined oil was kindly donated by Míča a Harašta (Prague, Czech Republic), and a refined, commercially available oil was purchased from Cannaderm (Prague, Czech Republic). The non-ionic surfactants Span 80 (Sorbitane monooleate, HLB 4.3), Span 85 (Sorbitane trioleate, HLB 1.8), Tween 80 (Polyoxyethylenesorbitan monooleate, HLB 15), and Tween 85 (Polyoxyethylenesorbitan monooleate, HLB 11) were supplied by Sigma-Aldrich (Steinheim, Germany) and were used without further purification.

### 3.2. Microorganisms

The test microorganisms, including gram-positive and gram-negative strains, were obtained from the Czech Collection of Microorganisms (CCM, Czech Republic). The bacteria were selected to represent the major spoilage classes. Tests utilized gram-positive *Bacillus subtilis*, subsp. *subtilis* CCM 2216, *Bacillus cereus* CCM 2010, *Enterococcus faecalis* CCM 4224, *Micrococcus luteus* CCM 732, *Staphylococcus aureus*, subsp. *aureus* CCM 3953, and gram-negative *Citrobacter freundii* CCM 7187, *Escherichia coli* CCM 3954, *Proteus vulgaris* CCM 1799, *Pseudomonas aeruginosa* CCM 3955, and *Serratia marcescens*, subsp. *marcescens* CCM 303. All strains were maintained on nutrient agar (5 g L^−1^ peptone, 5 g L^−1^ NaCl, 1.5 g L^−1^ beef extract, 1.5 g L^−1^ yeast extract, 15 g L^−1^ agar; from Hi-Media Laboratories Bombay, India) and were sub-cultured onto fresh media every two weeks. The initial test inocula of the microorganisms were prepared from 24 h cultures. Each bacterial suspension was adjusted by dilution with a nutrient broth to 5 × 10^8^ CFU mL^−1^.

### 3.3. Characterization of Hemp Seed Oil

Basic characteristics in terms of iodine, saponification, and acid value were determined using respective methods described elsewhere [42]. Fatty acid methyl esters (FAME) were prepared by transesterification with KOH in methanol. In brief, oil was mixed with methanolic potassium hydroxide (1 M), boiled for 30 min and cooled to room temperature. Hexane and aqueous sodium chloride solution were added, and the organic, upper layer was separated for gas chromatography (GC) analysis. The composition of fatty acids was determined using GC on a Shimadzu GC-14A device equipped with an flame ionization detector (FID). A capillary DB-WAX column (30 m × 0.25 mm, Agilent, Santa Clara, CA, USA) was used. The temperature program employed was as follows: the column temperature was programmed at 110 °C and maintained for 3 min, then the temperature was raised to 220 °C at a rate of 15 °C/min, and an isothermal step followed at this temperature for 10 min. The temperatures for the injector and detector were set at 225 °C and 230 °C, respectively. Identification of the fatty acids present was carried out using the FAME SUPELCO 37 Component FAME Mix (Sigma Aldrich, Steinheim, Germany) standard. The content of the respective FAME in oil was expressed in percent by applying an internal normalization procedure.

### 3.4. Preparation of Emulsions

Two different methods were carried out to prepare the emulsions: high-energy emulsification and low-energy, phase-inversion emulsification (EIP). The oil-to-water (O/W) ratio of 5/95 (*w/w*) was employed. Suitable pairs of Spans and Tweens (Table 3) at the amounts of 5 and 10 wt % were used to formulate emulsions with a hydrophilic-lipophilic balance (HLB) ranging from 6 to 10. The required HLB was calculated using the following formula:

(1) $$HLB=w_{1}\times HLB_{1}+w_{2}\times HLB_{2}$$

wherein *w*_1_ and *w*_2_ represent weight fractions of the emulsifiers utilized with *HLB*_1_ and *HLB*_2_, respectively. High-energy emulsification was performed on an Ultra Turrax T 25 device (IKA, Staufen, Germany). Appropriate amounts of hemp seed oil (5 wt %), a suitable pair of emulsifiers (5 or 10 wt %), and distilled water (add to 100 wt %) were heated in a test tube to 70 °C and homogenized immediately at 13,400 rpm for 15 min. The low-energy, phase-inversion procedure went as follows. Both the water and oil phases were heated to 70 °C and maintained at this temperature. The aqueous phase with a dissolved, water-soluble surfactant (Tween) was added drop-wise to the oil phase, which contained the oil-soluble surfactant (Span) and hemp seed oil. A constant stirring rate of 1050 rpm was utilized over a duration of 30 min. Homogenization was performed using an RZR Heidolph homogenizer (Heidolph Instruments GmbH & Co. KG, Schwabach, Germany).

### 3.5. Particle Size Measurements

Particle size, particle size distribution, and the polydispersity index (PDI) were determined by dynamic light scattering (DLS) on a Zetasizer Nano ZS instrument (Malvern Instruments, Malvern, UK). Measurements for the hydrodynamic radii of emulsion droplets were expressed as intensity-weighted z-average diameters (nm). Analyses were carried out at a scattering angle of 90° at the temperature of 25 °C. The stability of the emulsions was evaluated at regular time intervals by measuring the particle size under different storage conditions (25 and 4 °C).

### 3.6. Antibacterial Testing

The oil samples and their emulsions were screened for the antibacterial activity they exhibited against common pathogenic bacteria by utilizing the disk diffusion and broth microdilution methods.

Disk diffusion method: Suspensions of each tested microorganism (100 μL) were spread on Mueller-Hinton sterile agar plates (Hi-Media Laboratories, Bombay, India). Sterile paper discs of 6 mm diameter, soaked with 5 μL of sample (hemp seed oil or emulsions), were placed on surfaces of the agar plates. As a reference, sample discs soaked with emulsions absent of hemp seed oil were utilized. Antibacterial activity was evaluated by measuring the diameter of the inhibition zone in mm after 24 h of incubation at 30 °C (*Bacillus subtilis* subsp. *subtilis*; *Pseudomonas aeruginosa*; and *Bacillus cereus*) or 37 °C (the remaining bacteria) and expressed in mm. In order to calculate the inhibition zone, the diameter of the paper disc was subtracted from the diameter of the inhibition zone.

Broth microdilution method: Under sterile conditions, 20 µL of bacterial suspension and 200 µL of a sample (containing hemp seed oil or a prepared emulsion) were pipetted into each well of the 96-well sterile microplate. Nutrient agar inoculated with bacterial suspension was employed as a positive reference. Emulsions, absent of hemp seed oil were used as a negative reference. Plates were then incubated for 30 min at 30 °C. After incubation, 100 µL of each individual suspension present in a respective well was spread over the surface of an agar plate and incubated again for 24 h at either 30 °C or 37 °C, depending on the bacteria used.

### 3.7. Statistical Analysis

The sizes of emulsion particles (DLS), iodine, saponification, and acid values as well as fatty acid composition (GC) of the hemp-seed oils were analysed at least in triplicate (*n* = 3); means and standard deviations were calculated in accordance with the Dean-Dixon method. Correspondingly, antibacterial testing (disk diffusion and broth microdilution methods) was conducted in triplicate and the Dean-Dixon method was utilized to calculate the means and standard deviations. The T-test was applied to determine statistical differences between the individual samples (Statistica, StatSoft, Inc., Tulsa, OK, USA). The *p* values of ≤0.05 were considered statistically significant.

## 4. Conclusions

This study has shown that emulsions of bioactive hemp seed oil can be formulated by employing pairs of non-ionic surfactants (Span, Tween) under appropriate conditions. It was found that the ability to form stable emulsions of small, initial particle size is primarily dependent on the given method of preparation and the HLB value. The low-energy method was suitable for producing emulsions without a high-energy input, but these systems turned out to be unstable due to the large, initial droplet sizes (502 ± 22 nm to 1050 ± 29 nm). However, high-energy homogenization produced nanoemulsions with fine droplets (151 ± 1 nm to 209 ± 5 nm), also supporting the stability of the emulsions. Regarding the influence of oil type on the formulations, emulsions containing refined and unrefined hemp seed oil performed similarly when using the high-energy method but differed when low-energy emulsification was employed. Testing for antibacterial properties by the disk diffusion and broth dilution methods confirmed the activity of the hemp seed oil utilized herein, although there was no sign of the enhancement that had been anticipated in the capability of the oil to act against bacteria via encapsulation in the emulsions. Regardless of this fact, the results suggest that such formulated emulsions could serve to fortify foodstuffs, as hemp seed oil is an exceptionally rich source of essential fatty acids, and the n-6 to n-3 fatty acid ratio in the oil stands at 3:1, which is considered optimal for human dietary purposes.

## Figures and Tables

**Table 1:** Composition of fatty acids (g/100 g) in unrefined and refined hemp seed oils determined by gas chromatography (GC).

Fatty Acid	Concentration (g/100 g)	
	Unrefined Oil	Refined Oil
Myristic	0.04 ± 0.03	n.d.
Palmitic	5.9 ± 0.27	6.2 ± 0.13
Palmitoleic	0.1 ± 0.00	0.1 ± 0.02
Stearic	2.2 ± 0.04	2.4 ± 0.07
Oleic	9.0 ± 0.15	12.1 ± 0.03
Linoleic	55.3 ± 0.12	57.3 ± 0.03
gamma-linolenic	4.4 ± 0.03	3.0 ± 0.02
alpha-linolenic	20.3 ± 0.03	16.7 ± 0.04
Arachidic	1.7 ± 0.04	1.0 ± 0.04
Eikosanoic	0.7 ± 0.05	0.8 ± 0.00
Eikosenoic	0.4 ± 0.02	0.4 ± 0.02
Eikosadienoic	0.1 ± 0.01	n.d.

n.d.: not determined.

**Table 2:** Inhibitory effect of hempseed oils (mean ± SD, *n = 3*) against most common pathogenic bacteria, expressed as diameter of the inhibition zone in mm (diameter of the disc was subtracted from the total size of inhibition zone). Means within a line with the same superscript differ significantly (*p* ≥ 0.005).

Bacterial Strain		Inhibition Zone Size (Ø ± σ) mm	
		Unrefined Oil	Refined Oil
*G^+^*	*Bacillus cereus* CCM 2010	2.3 ± 0.6 ^a^	0.0 ± 0.0 ^a^
	*Bacillus subtilis* subsp. *subtilis* CCM 2216	2.3 ± 1.8 ^a^	0.0 ± 0.0 ^a^
	*Micrococcus luteus* CCM 732	3.3 ± 1.8	2.7 ± 1.2
	*Staphylococcus aureus* subsp. *aureus* CCM 3953	3.0 ± 0.0	3.0 ± 1.2
*G^−^*	*Citrobacter freundii* CCM 7187	2.3 ± 0.6 ^a^	0.0 ± 0.0 ^a^
	*Enterococcus faecalis* CCM 4224	2.3 ± 0.6	2.7 ± 0.6
	*Escherichia coli* CCM 3954	0.3 ± 0.6	0.0 ± 0.0
	*Salmonella enterica* subsp. *enterica* ser. Enteritidis CCM 4420	3.0 ± 1.8	2.0 ± 1.2
	*Serratia marcescens* subsp. *marcescens* CCM 303	2.7 ± 0.6	0.7 ± 1.2
	*Pseudomonas aeruginosa* CCM 395	1.7 ± 0.6	2.3 ± 0.6

**Table 3:** Mixtures on non-ionic surfactants used for obtaining the chosen HLB values; design of the study used for the preparation of hemp seed oil emulsions. Amounts of surfactants are given for their 5 wt % contents in 100 g of emulsions.

Amount of Surfactants (g)				HLB of the Mixture (*Tween/Span Ratio*)
Tween 80 (HLB 15)	Span 80 (HLB 4.3)	Tween 85 (HLB 11)	Span 85 (HLB 1.8)	
0.794	4.206	-	-	6 *(0.19)*
1.262	3.738	-	-	7 *(0.34)*
-	-	2.826	2.174	7 *(1.30)*
1.729	3.271	-	-	8 *(0.52)*
-	-	3.370	1.630	8 *(2.07)*
2.196	2.804	-	-	9 *(0.78)*
-	-	3.913	1.087	9 *(3.60)*
2.664	2.336	-	-	10 *(1.14)*
